# Development and Validation of a Small for Gestational Age Screening Model at 21–24 Weeks Based on the Real-World Clinical Data

**DOI:** 10.3390/jcm12082993

**Published:** 2023-04-20

**Authors:** Jing Gao, Zhongzhou Xiao, Chao Chen, Hu-Wei Shi, Sen Yang, Lei Chen, Jie Xu, Weiwei Cheng

**Affiliations:** 1 International Peace Maternity and Child Health Hospital, School of Medicine, Shanghai Jiao Tong University, Shanghai 200030, China; 2 Shanghai Key Laboratory of Embryo Original Disease, Shanghai 200040, China; 3 Shanghai Municipal Key Clinical Specialty, Shanghai 200030, China; 4 Shanghai Artificial Intelligence Laboratory, Shanghai 200030, China

**Keywords:** small for gestational age, fetal growth restriction, predictive model, risk screening, ultrasonography

## Abstract

Background: Small for gestational age (SGA) is a condition in which fetal birthweight is below the 10th percentile for the gestational age, which increases the risk of perinatal morbidity and mortality. Therefore, early screening for each pregnant woman is of great interest. We aimed to develop an accurate and widely applicable screening model for SGA at 21–24 gestational weeks of singleton pregnancies. Methods: This retrospective observational study included medical records of 23,783 pregnant women who gave birth to singleton infants at a tertiary hospital in Shanghai between 1 January 2018 and 31 December 2019. The obtained data were nonrandomly classified into training (1 January 2018 to 31 December 2018) and validation (1 January 2019 to 31 December 2019) datasets based on the year of data collection. The study variables, including maternal characteristics, laboratory test results, and sonographic parameters at 21–24 weeks of gestation were compared between the two groups. Further, univariate and multivariate logistic regression analyses were performed to identify independent risk factors for SGA. The reduced model was presented as a nomogram. The performance of the nomogram was assessed in terms of its discrimination, calibration, and clinical usefulness. Moreover, its performance was assessed in the preterm subgroup of SGA. Results: Overall, 11,746 and 12,037 cases were included in the training and validation datasets, respectively. The developed SGA nomogram, comprising 12 selected variables, including age, gravidity, parity, body mass index, gestational age, single umbilical artery, abdominal circumference, humerus length, abdominal anteroposterior trunk diameter, umbilical artery systolic/diastolic ratio, transverse trunk diameter, and fasting plasma glucose, was significantly associated with SGA. The area under the curve value of our SGA nomogram model was 0.7, indicating a good identification ability and favorable calibration. Regarding preterm SGA fetuses, the nomogram achieved a satisfactory performance, with an average prediction rate of 86.3%. Conclusions: Our model is a reliable screening tool for SGA at 21–24 gestational weeks, especially for high-risk preterm fetuses. We believe that it will help clinical healthcare staff to arrange more comprehensive prenatal care examinations and, consequently, provide a timely diagnosis, intervention, and delivery.

## 1. Introduction

The term small for gestational age (SGA) is exclusively used to describe newborns with birth weights below the 10th percentile. Its prevalence is 3–10% and 6.5% worldwide and in China, respectively [1]. Similarly, infants with fetal growth restriction (FGR) fail to achieve their full in utero growth potential and have a pathological condition whose causes can be broadly classified into maternal, fetal, and placental causes [2]. Notably, SGA or FGR can be mostly attributed to suboptimal uterine–placental perfusion and fetal nutrition, increasing the risks of perinatal morbidity and mortality and leading to adverse effects in later life (e.g., cognitive, behavioral, and socioemotional dysfunction in childhood, and metabolism and cardiovascular diseases in adulthood) [3]. Exact evaluation of FGR during antenatal care is difficult; hence, SGA is commonly used as a proxy for screening suspected FGR pregnancies. Indeed, the terms SGA and FGR are often used interchangeably [4]. The traditional method for diagnosing SGA neonates is maternal abdominal palpation; however, its performance is poor as its prediction accuracy is only 21% [5]. Recently, sonographic fetal biometry and Doppler velocimetry evaluation of the umbilical artery (UA) have been reported to improve the diagnostic accuracy of SGA. A Cochrane review published in 2017 reported that UA Doppler imaging in high-risk pregnancies could reduce the rate of perinatal child deaths by 29% [6]; however, this imaging is performed in the third trimester. Early detection will enable improved outcomes in these fetuses as it allows for the adjustment of follow-up and delivery times. Alexandros et al. developed a simple model to predict FGR and SGA by combining the predictors used in the first and second trimesters. In a previous study, the detection rate of 10% false positives was suboptimal for FGR (59.6%) and SGA (41.8%) [7]. Thus, some scientists are striving to achieve higher accuracy using novel predictors, such as serum placental growth factor, soluble fms-like tyrosine kinase-1 (sFlt-1), pregnancy-associated plasma protein-A (PAPP-A), and nuchal fold thickness, which are not routinely assessed in clinical practice [8].

This retrospective large cohort study aimed to establish an accurate widely applicable screening tool for SGA at 21–24 gestational weeks of singleton pregnancies. Accurately identifying fetuses at high risk for developing SGA can aid in their antenatal management and allow for timely interventions to reduce associated morbidity and mortality.

## 2. Materials and Methods

This study was conducted in accordance with the principles of the Declaration of Helsinki. The requirement for informed consent was waived by the Ethics Committee of the International Peace Maternity and Child Health Hospital owing to the retrospective nature of the study. Moreover, the study was reported in accordance with the Transparent Reporting of a multivariable prediction model for Individual Prognosis or Diagnosis (TRIPOD) statement [9]. The official TRIPOD checklist is presented in Supplemental Table S1.

### 2.1. Study Population

**Figure 1:**
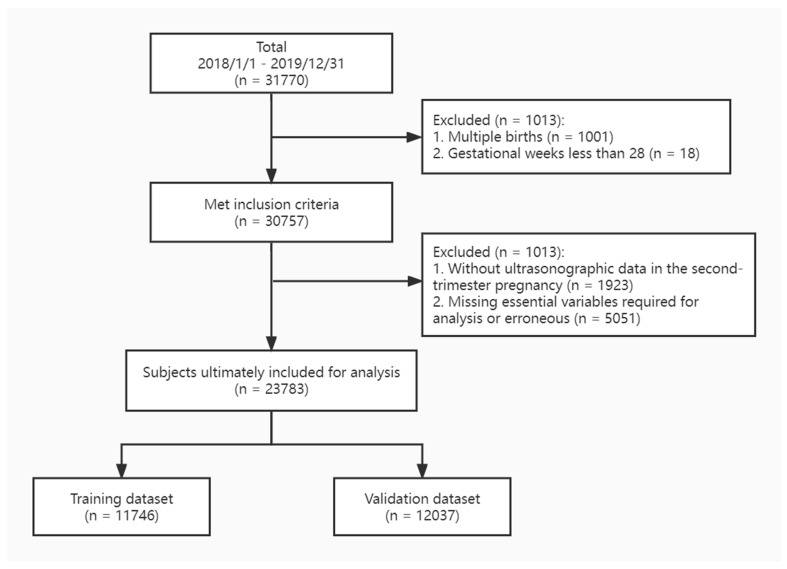
Flowchart illustrating pregnant women included in the study.

For this retrospective study, we collected data from the digital medical records system of the International Peace Maternity and Child Health Hospital—a tertiary-care hospital in Shanghai—between 1 January 2018 and 31 December 2019. The inclusion criteria for the present study were as follows: (1) a singleton pregnancy; (2) gestation ≥ 28 weeks; and (3) no severe fetal chromosomal or structural abnormalities. After data screening, 30,757 individuals were included in the study (Figure 1).

### 2.2. Variables Measurements

Using studies and reviews in the relevant literature, we searched for SGA variables that can be easily evaluated in different settings by examiners with different levels of clinical experience and as a part of routine examinations during pregnancy. In China, ultrasound screening of fetal malformations is currently performed at 21–24 gestational weeks. Therefore, we used maternal data (including demographic characteristics, blood glucose levels, and blood lipid levels) and results of sonographic examinations (first ultrasound screening of fetal malformations at 21–24 weeks of gestation) as variables in our model. Further, we collected data regarding the mother’s and father’s demographic characteristics, medical history, and reproductive history at the first antenatal visit during 9–13 weeks of gestation. Moreover, data regarding maternal height, weight, gravidity, parity, and educational level were obtained via face-to-face interviews. The pre-pregnancy body mass index (pre-pregnancy BMI) was calculated by dividing the pre-pregnancy weight (kg) by the square of the pre-pregnancy height (m^2^). The obtained BMI values were classified into four levels using the cutoff for Asian adults, as proposed by the World Health Organization [10]: <18.5 kg/m^2^, underweight; 18.5–24.9 kg/m^2^, normal weight; 25.0–29.9 kg/m^2^, overweight; and 30 kg/m^2^, obesity. Gestational age was derived from sonographic measurement of the fetal crown–rump length or biparietal diameter. Maternal fasting lipid serum samples were obtained in the first trimester (9–14 weeks), collected in 10-mL vacutainer tubes, and centrifuged. The following laboratory indices were calculated: triglycerides (TG), total cholesterol, high-density lipoprotein, and low-density lipoprotein. Furthermore, values of blood pressure (systolic blood pressure and diastolic blood pressure) and glucose index (fasting plasma glucose [FPG], one-hour glucose [GLU-1H], two-hour glucose [GLU-2H], and glycosylated hemoglobin [HbA1c]) were recorded on the day of the 75 g oral glucose tolerance test between 24 and 28 weeks of gestation. Moreover, we used the Phillips IU22 ultrasound with a probe frequency of 3.5 MHz. Pregnancy was determined after the woman was placed in a prone position, slightly filling her bladder and controlling her respiratory intensity. The following parameters were measured in accordance with standardized recommendations [11]: biparietal diameter (BPD), head circumference (HC), abdominal circumference (AC), humerus length (HL), transverse trunk diameter (TTD), anteroposterior trunk diameter (APTD), and max amniotic fluid volume (AFV). UA Doppler flow indices included the following: systolic/diastolic ratio (S/D), pulsatility index (PI), and resistance index (RI). Moreover, we evaluated the placental thickness, placental location (normal, placenta previa, or low-lying), placental sinusoids, single umbilical artery (SUA), and velamentous cord insertion placenta. All examiners were senior doctors with standard training and >5 years of experience in obstetric ultrasonography.

### 2.3. Outcomes

Each neonate’s birthweight (in grams) was routinely measured by registered midwives using an electronic weighing scale within 30 min of delivery. SGA was defined as a birth weight below the 10th percentile as per the Chinese neonatal birth weight curve for different gestational ages [12].

### 2.4. Data Processing

The data were collected and stored in Microsoft Office Excel^®^ 2019 (Microsoft Corporation, Santa Rosa, CA, USA); subsequently, they were exported to R software version 4.2.1 (R Core Team, Vienna, Austria) for data preprocessing. Regarding obstetric and ultrasonographic characteristics, the proportions of missing data varied and are summarized in Supplemental Table S4. Based on our clinical experience, we assumed that the data were missing at random (MAR). Thus, multivariate imputation by chained equations was performed for these missing values [13]. Subsequently, a tiny proportion (<1%) of the missing values remained. We excluded these values to ensure data integrity. Further, sensitivity analyses were performed to determine whether the imputation values were robust and whether the assumption of MAR was valid. The results provided approximate measurements, which were comparable (Supplemental Materials).

### 2.5. Statistical Analysis

First, preliminary statistical analyses were performed, including the normality test and the correlation analysis of covariates. In the normality test, we assessed whether the data followed a normal distribution using a combination of the Kolmogorov–Smirnov test and QQ plots. Medians and interquartile ranges (IQRs) were used to present continuous variables, whereas counts and percentages were used to present categorical variables. The Wilcoxon rank sum test was used for comparisons of continuous variables between the groups. The chi-squared or Fisher’s exact test was used for categorical variables, as appropriate. Two-sided *p*-values of <0.05 were considered significant. All statistical analyses were performed using R version 4.2.1 (23 June 2022).

According to the Transparent Reporting of a multivariable prediction model for Individual Prognosis or Diagnosis (TRIPOD) statement, data were nonrandomly divided into a training set (1 January 2018 to 31 December 2018) and a validation set (1 January 2019 to 31 December 2019) in terms of the year of data collection. Notably, this is a better design for evaluating model performance than random division because it allows for evaluating nonrandom variation between the two data sets. First, we conducted a univariate logistic regression analysis to acquire information about the relationship between each potential determinant and SGA. All statistically significant covariates were selected for a subsequent multivariate logistic regression analysis. The multivariate logistic regression analysis with a backward stepwise selection (using Akaike’s information criterion [AIC]) was then used for the training set. Subsequently, multicollinearity was evaluated by assessing the value of the variance inflation factor (VIF). Thereafter, a nomogram was created using the results of the multivariate logistic regression analysis.

The calibration curve was used to assess the calibration ability of the nomogram in addition to the Hosmer–Lemeshow test. Further, we measured the area under the receiver operating characteristic curve (AUC) to quantify the discrimination performance of the nomogram. Bootstrapping validation with 1000 chosen resamples was used to validate AUC and its 95% confidence interval, both internally (training set) and externally (validation set), as well as the calibration curve. The model’s predictive performance after bootstrapping could be applied to other similar populations to some extent. Regarding its usefulness in clinical practice, a decision curve analysis (DCA) was used to investigate the overall benefits in patients with pre-specified threshold probabilities in the validation dataset [14]. Further, we compared the prediction model with two reasonable clinical strategies: intervention for all and intervention for none.

Focusing on the cases of preterm births (gestational age < 37 weeks), a post hoc analysis was performed to compare the differences between cases that were successfully predicted (true positives) and cases that were not predicted (false negatives) using the Wilcoxon test.

## 3. Result

### 3.1. Baseline Demographic, Blood Testing, and Ultrasonographic Characteristics of Pregnant Women

This study enrolled 23,783 pregnant women, including 11,746 who gave birth in 2018 (training dataset) and 12,037 who gave birth in 2019 (validation dataset). Figure 1 shows the flow chart of the inclusion of the study population. We compared the differences between the SGA and non-SGA groups within the two datasets and found no statistically significant differences in SGA prevalence between the datasets (4.07% vs. 4.32%; *p* = 0.486).

The baseline demographic characteristics of pregnant women are presented in Table 1. With a similar distribution, we found an inconsistency in the significant differences in some characteristics, including age, father’s education level, and family history of diabetes or hypertension, and between the training and validation datasets; however, other characteristics did not differ significantly. The median ages of mothers and fathers were similar (31 [IQR: 28–34] and 32 [IQR: 29–35], respectively). Moreover, in both datasets, the education levels of mothers and fathers were comparable, and most of them had a bachelor’s degree. However, the two groups (SGA or non-SGA) significantly differed in terms of BMI, GA, father’s age, gravidity, and parity.

Table 2 shows the medical characteristics of pregnant women, including obstetric and ultrasonographic results. Although we found some inconsistencies, there were no significant differences in clinical features between the two datasets, regardless of the group (SGA or non-SGA), further proving the validity of the split datasets. In contrast, there were significant differences between the two groups in terms of BPD, AC, HC, FL, HL, TTD, APTD, AFV, placental thickness, umbilical artery systolic/diastolic ratio (UA-S/D), UA (PI), UA (RI), FPG, and GLU-2H (*p* < 0.05).

### 3.2. Model Development: Univariate and Multivariate Analysis

Among the covariates included, 24 potential risk factors of 38 variables were excluded by univariate analysis of the training dataset based on a *p*-value of <0.05. Furthermore, the 24 variables were used in the backward multivariate logistic regression analysis, resulting in 12 remaining variables: age, gravidity, parity, BMI, gestational age, SUA, AC, HL, abdominal APTD, UA-S/D, TTD, and FPG. Owing to the strong correlation between TTD and APTD and a strong VIF value, the variable TTD was excluded based on the Delphi method, which involves the consultation of expert-based opinions to make informed decisions [15]. Furthermore, we developed a logistic regression incorporating the above factors. The odds ratio (OR) and its 95% confidence interval for each factor are shown in Table 3. These data indicate that the independent predictors for SGA were advanced maternal age women (OR: 1.23), SUA (OR: 3.25), and UA-S/D (OR: 1.4). The results of the univariate analysis are provided in the Supplemental Materials.

### 3.3. Model Performance and Validation

**Figure 2:**
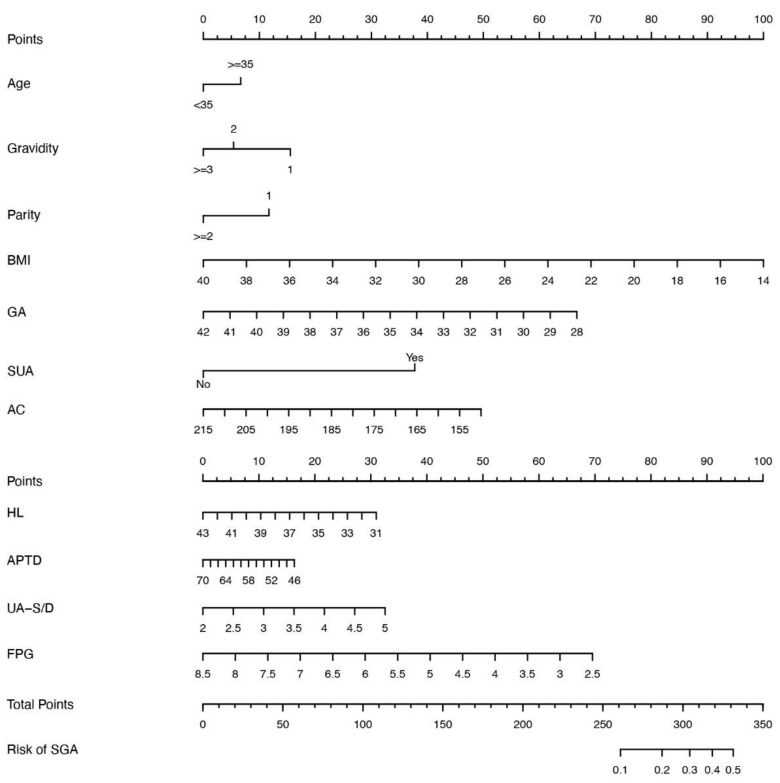
Nomogram model for predicting the risk of small for gestational age in the mid-trimester pregnancy. BMI, body mass index; GA, gestational age; SUA, single umbilical artery; AC, abdominal circumference; humeral length APTD, anteroposterior trunk diameter; UA-SD, Umbilical artery systolic/diastolic ratio; FBG, fasting blood glucose; SGA, small for gestational age.

**Figure 3:**
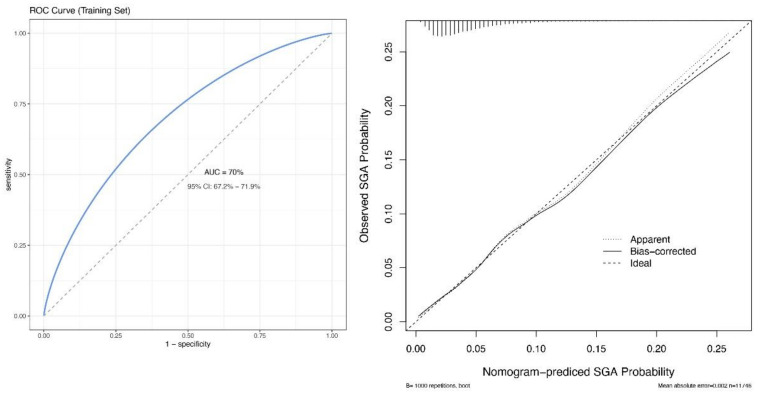
Receiver operating characteristic and calibration curves in the training dataset. ROC, receiver operating characteristic; SGA, small for gestational age.

A nomogram was created to quantify and illustrate the prediction model based on the above-mentioned 12 predictors to predict the risk of SGA (Figure 2). Each predictor was assigned a score based on the characteristics of each pregnant woman. The total score was then calculated to obtain the risk probability [16]. The prediction nomogram achieved acceptable performance, with an AUC of 0.70 (95% CI: 0.67–0.72) based on the 2018 data extracted from the internal validation dataset. This finding was confirmed to be reliable using a bootstrapping method with binormal smoothing. A perfect concordance was found between observation and prediction (Figure 3). Moreover, the Hosmer–Lemeshow test yielded a nonsignificant result (*p* = 0.84), suggesting no evidence of a poor fit.

**Figure 4:**
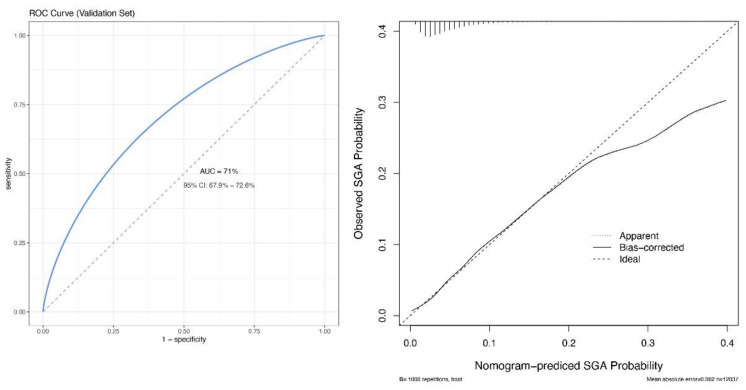
Receiver operating characteristic and calibration curves in the validation dataset. ROC, receiver operating characteristic; SGA, small for gestational age.

The discriminative capability of the model slightly improved in the external validation dataset (Figure 4), with an AUC of 0.71 (95% CI: 0.68–0.73). Moreover, the calibration curve of the nomogram indicated good agreement between the observed and predicted probability in the external validation dataset, except for a slight departure on the upper tail of the curve (Figure 4). Furthermore, the Hosmer–Lemeshow test provided a nonsignificant result (*p* = 0.39).

### 3.4. Model Cost-Benefit Analysis (DCA)

**Figure 5:**
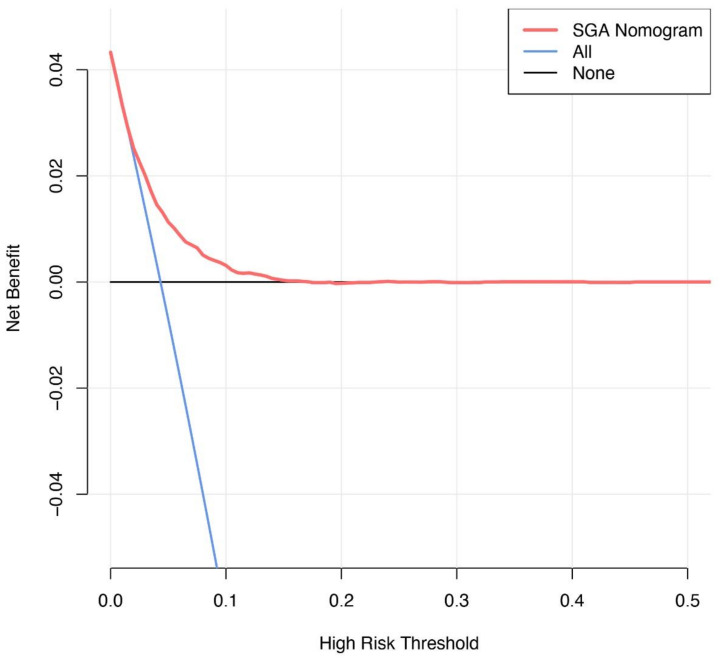
Decision curve analysis for the small for gestational age (SGA) prediction model.

The DCA for the SGA prediction model was performed (Figure 5). The net benefit was calculated for all threshold probabilities, ranging from 0 to 1. The decision curve of our model indicated that the use of the nomogram would be more beneficial than the use of intervention for all and intervention for none scheme to predict SGA if the chosen threshold probability is between 4% and 15%.

### 3.5. The Performance of the Preterm Subgroup

**Figure 6:**
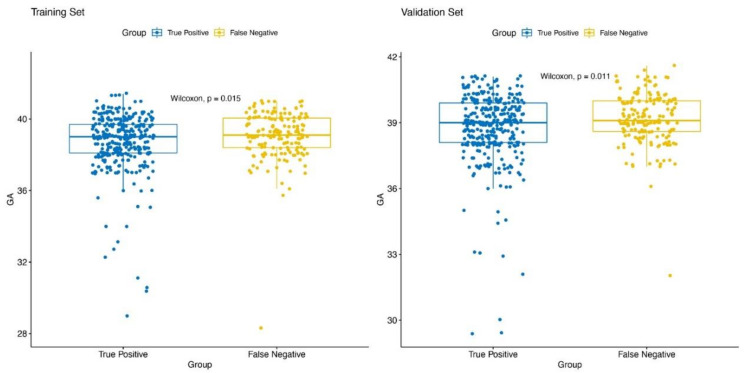
Box-and-whisker plots of gestational age between small for gestational age fetuses truly predicted by our model and not predicted by our model.

Among the true SGA fetuses, 33 and 40 in the training and validation datasets were born preterm, respectively. Of them, 27 (81.8%) and 36 (90.0%) were successfully predicted using the model (average prediction rate: 86.3%). Regarding the comparison between the SGA fetuses truly predicted by our model and those not predicted by our model, the mean gestational ages of truly predicted infants were significantly lower in both training (38.73 vs. 39.08, *p* < 0.05) and validation (38.78 vs. 39.20, *p* < 0.05) datasets. This indicates that the model has the advantage of accurately predicting SGA at a relatively small gestational age. To better illustrate these differences, box-and-whisker plots are shown in Figure 6.

## 4. Discussion

SGA is a complex and multifactorial disorder that affects fetal development and often results in stillbirth or other perinatal complications. Early screening to predict the likelihood of FGR in fetuses is expected to help with timely diagnosis through intensive follow-up or detailed examination [17]. Based on the clinical data from a large cohort of pregnant women, our study retrospectively identified the risk factors for SGA and developed a good predictive model advanced to 21–24 weeks of gestation.

Considering the heterogeneous causes of SGA, we used both maternal information and the results of the sonographic examination in utero at 22–25 weeks of gestation as variables in our model. Furthermore, using logistic regression analyses, we identified 12 risk factors: age, gravidity, parity, BMI, gestational age, SUA, AC, HL, abdominal APTD, UA-S/D, TTD, and FPG. The values of these indicators are available in routine medical records, indicating that our model is widely applicable. Various studies have reported predictive models with novel biochemical markers, such as PAPP-A, alfa-fetoprotein, AFP, and human chorionic gonadotropin, which are known to be partly associated with placental function. For example, Sotiriadis et al. [18] combined first- and second-trimester markers, including PAPP-A, for establishing their model. The detection rate for 10% false positives was good for late FGR (78.6%). However, the causes and mechanisms of these novel markers remain unconfirmed [19]. Unless the pregnancies are considered high-risk, the use of these novel markers for all pregnant women may be considered excessive medical care.

The results of the present study indicated that gravida and parity are negatively associated with SGA; this finding is similar to that of previous studies. Li Lin et al. [20] conducted a retrospective study of Chinese individuals and found that multiparity was associated with a reduced risk of LBW (aRR = 0.74, 95% CI: 0.72–0.77) and SGA (aRR = 0.67, 95% CI: 0.66–0.69) compared with nulliparity. Furthermore, a meta-analysis of 41 studies suggested that nulliparous mothers have an 89% increased risk of SGA [21]. This can be attributed to less uteroplacental blood flow and smaller uterine cavities in women who have never given birth or conceived before [22]. Results of a previous study that used uterine artery Doppler velocimetry revealed that the prevalence of uterine artery notches is significantly higher in nulliparous women, suggesting a higher uteroplacental blood impedance to flow [23]. Furthermore, pregnancy at an advanced age (≥35 years) was identified as an independent risk factor for SGA. Another study of 137,791 women reported that the risk of SGA increased with maternal age and that the risk increased earlier in nulliparous women [24]. Interestingly, in the subgroup analysis by Palatnik et al. [25], nulliparous women aged ≥30 years (but not multiparous women) and all women aged ≥40 years had a high risk of developing SGA. Delayed childbearing has been a growing trend in many countries over the last few decades [26], increasing the number of nulliparous women at an advanced age. Therefore, monitoring and treating primiparous women aged >35 years is a challenge for all involved in the fight to minimize the consequences of SGA fetuses.

To the best of our knowledge, there is no effective treatment available to reverse the course of FGR, except for delivery [27]. According to the 2020 Society for Maternal-Fetal Medicine Consult Series, delivery of an FGR fetus is recommended after 37 weeks of gestation based on the percentile of the estimated fetal weight [28]. Moreover, earlier delivery is indicated in cases of absent or reverse UA flow owing to the high impedance uteroplacental perfusion [29]. Furthermore, poor maternal conditions, such as hypertensive disease of pregnancy, cardiac disease, and immune system disease, could result in preterm birth, indicating that the earlier the delivery, the worse the intrauterine environment and/or maternal condition. Our model achieved a relatively acceptable accuracy of SGA prediction and demonstrated good performance in identifying SGA pregnancy with a high risk of preterm birth.

Our nomogram model can serve as a practical tool for the clinical screening of SGA. Once it indicates the risk of SGA in pregnant women, it suggests that the pregnant woman needs more attention. Notably, it has been recommended to shorten the visit intervals. In addition, finding possible etiologies, such as maternal malnutrition, poor weight gain, smoking, congenital fetal infections, and fetal genetic or structural disorders, should be the first goal. However, in most cases, doctors often fail to identify the etiology. Doppler sonography plays a significant role in assessing fetal developmental conditions and trends [30]. Currently, the middle cerebral artery and ductus venosus Doppler, except for UA Doppler, represent chronic fetal hypoxia, thereby helping to determine the timing of delivery. Cardiac and aortic isthmus Doppler allows the evaluation of heart functionality with the deterioration of FGR [31]. MacDonald et al. [32] reported a novel predictor, the ratio of the middle cerebral artery to the UA pulsatility indices (CPUR), which indicated the strongest association with indicators of placental insufficiency. However, Rial Crestelo et al. [33] indicated that the added value of CPUR at 33 weeks of gestation for detecting defective growth is poor in nonselected pregnancies.

Our study has some limitations. Although the overall sample size was considerable, all participants were recruited from the same obstetric hospital, principally covering low to moderate risk pregnant women. This cohort may not completely represent obstetric practice in the community. Second, the accuracy of our model was not optimal. One of the reasons for this could be the limited number of variables. The policy at our institution is not to reveal the fetal sex before delivery; hence, we did not select fetal sex as one of the variables. Third, in our retrospective study, we could not collect relevant information regarding gestational hypertension in the mid-trimester. Some studies reported that 30–40% of cases of FGR are complicated by placental function impairment due to pre-eclampsia, chronic hypertension, and gestational hypertension [34]. The predictive power of our model can be improved if we collect more data regarding hypertension changes, drug treatment, complications, and other variables. Further studies are warranted to include more appropriate influencing factors and use prospective design to optimize our model.

Our SGA model focused on the issue within the medical framework of Prediction, Prevention, Personalization, and Precision, and it highlighted the significance of timely detection of high-risk pregnancies with high accuracy. In clinical practice, once the model indicates the possibility of SGA, obstetricians are advised to search for possible etiologies, shorten visit intervals, and seek detailed investigations (for example, blood pressure, urine protein, and ultrasound Doppler examination). This will guide clinical healthcare workers to arrange for more prenatal care examinations and, thus, enable timely diagnosis, intervention, and delivery.

## 5. Conclusions

Our model represents a reliable screening tool for SGA at 21–24 weeks gestation, particularly for fetuses at high risk of preterm delivery. In fact, our model has a wide application for improving pregnancy outcomes and expectations for newborns and optimizing medical cost-efficiency.

## Figures and Tables

**Table 1:** Baseline characteristics.

	Training Set			Validation Set		
Characteristic	Non-SGA ^1^ (n = 11,259)	SGA ^1^ (n = 487)	*p*-Value ^2^	Non-SGA ^1^ (n = 11,516)	SGA ^1^ (n = 521)	*p*-Value ^2^
BMI	20.9 (19.5, 22.9)	20.0 (18.7, 21.6)	<0.001	20.9 (19.5, 22.6)	20.2 (18.7, 21.5)	<0.001
GA	39.1 (38.4, 40.0)	39.1 (38.1, 39.9)	0.002	39.1 (38.6, 40.0)	39.0 (38.3, 39.9)	0.010
SBP	110 (103, 118)	110 (102, 119)	0.8	110 (103, 118)	109 (103, 116)	0.14
DBP	68 (63, 74)	69 (63, 75)	0.5	69 (63, 75)	68 (63, 74)	0.4
Age			0.046			0.3
<35	8820 (78%)	400 (82%)		9113 (79%)	422 (81%)	
≥35	2439 (22%)	87 (18%)		2403 (21%)	99 (19%)	
Hus age			0.009			0.018
<35	7788 (69%)	364 (75%)		8386 (73%)	404 (78%)	
≥35	3471 (31%)	123 (25%)		3130 (27%)	117 (22%)	
Edu			>0.9			0.6
Bachelor	5865 (52%)	256 (53%)		5708 (50%)	252 (48%)	
Above	2264 (20%)	101 (21%)		2206 (19%)	99 (19%)	
Below	2778 (25%)	114 (23%)		2546 (22%)	113 (22%)	
Unknown	352 (3.1%)	16 (3.3%)		1056 (9.2%)	57 (11%)	
Hus edu			0.6			0.030
Bachelor	5648 (50%)	255 (52%)		5427 (47%)	248 (48%)	
Above	2519 (22%)	111 (23%)		2433 (21%)	98 (19%)	
Below	2405 (21%)	91 (19%)		2280 (20%)	92 (18%)	
Unknown	687 (6.1%)	30 (6.2%)		1376 (12%)	83 (16%)	
Gravidity			<0.001			<0.001
1	5202 (46%)	296 (61%)		5655 (49%)	318 (61%)	
2	3384 (30%)	120 (25%)		3392 (29%)	133 (26%)	
≥3	2673 (24%)	71 (15%)		2469 (21%)	70 (13%)	
Parity			<0.001			<0.001
1	7584 (67%)	390 (80%)		8095 (70%)	432 (83%)	
≥2	3675 (33%)	97 (20%)		3421 (30%)	89 (17%)	
Smoking			0.6			0.4
No	10,831 (96%)	471 (97%)		10,371 (90%)	460 (88%)	
Yes	54 (0.5%)	3 (0.6%)		81 (0.7%)	4 (0.8%)	
Unknown	374 (3.3%)	13 (2.7%)		1064 (9.2%)	57 (11%)	
Alcohol			0.4			0.4
No	10,700 (95%)	469 (96%)		9765 (85%)	432 (83%)	
Yes	185 (1.6%)	5 (1.0%)		687 (6.0%)	32 (6.1%)	
Unknown	374 (3.3%)	13 (2.7%)		1064 (9.2%)	57 (11%)	
Family history of diabetes or hypertension			0.8			0.018
No	8583 (76%)	377 (77%)		7603 (66%)	313 (60%)	
Yes	2294 (20%)	94 (19%)		2317 (20%)	120 (23%)	
Unknown	382 (3.4%)	16 (3.3%)		1596 (14%)	88 (17%)	
Conception			>0.9			0.2
Natural	10,175 (90%)	441 (91%)		9705 (84%)	434 (83%)	
ART	789 (7.0%)	34 (7.0%)		841 (7.3%)	32 (6.1%)	
Unknown	295 (2.6%)	12 (2.5%)		970 (8.4%)	55 (11%)	

BMI, body mass index; GA, gestational age; SBP, systolic blood pressure; DBP, diastolic blood pressure; Hus age, husband’s age; Edu, education; Hus edu, husband’s education; SGA, small for gestational age. ^1^ Median (IQR); n (%). ^2^ Wilcoxon rank sum test; Pearson’s chi-squared test; Fisher’s exact probability test.

**Table 2:** Medical characteristics.

	Training Set			Validation Set		
Characteristic	Non-SGA ^1^ (n = 11,259)	SGA ^1^ (n = 487)	*p*-Value ^2^	Non-SGA ^1^ (n = 11,516)	SGA ^1^ (n = 521)	*p*-Value ^2^
Placental location			0.3			0.7
Normal	10,831 (96%)	475 (98%)		11,035 (96%)	503 (97%)	
Low-lying	192 (1.7%)	5 (1.0%)		197 (1.7%)	8 (1.5%)	
Previa	236 (2.1%)	7 (1.4%)		284 (2.5%)	10 (1.9%)	
Velamentous Placenta			>0.9			0.5
No	11,226 (99%)	486 (99%)		11,472 (100%)	518 (99%)	
Yes	33 (0.3%)	1 (0.2%)		44 (0.4%)	3 (0.6%)	
Placental sinusoids			0.7			0.4
No	11,205 (99%)	486 (99%)		11,443 (99%)	516 (99%)	
Yes	54 (0.5%)	1 (0.2%)		73 (0.6%)	5 (1.0%)	
SUA			0.046			0.2
No	11,230 (99%)	483 (99%)		11,479 (100%)	518 (99%)	
Yes	29 (0.3%)	4 (0.8%)		37 (0.3%)	3 (0.6%)	
BPD	56.0 (54.0, 58.0)	55.0 (53.0, 58.0)	<0.001	56.0 (54.0, 58.0)	55.0 (53.0, 57.0)	<0.001
AC	175 (169, 183)	171 (164, 178)	<0.001	174 (167, 180)	169 (163, 175)	<0.001
HC	199 (193, 206)	195 (189, 203)	<0.001	198 (193, 204)	195 (189, 201)	<0.001
FL	38.0 (37.0, 40.0)	38.0 (36.0, 39.0)	<0.001	38.0 (37.0, 40.0)	38.0 (36.0, 39.0)	<0.001
HL	36.0 (35.0, 38.0)	36.0 (34.0, 37.0)	<0.001	36.0 (35.0, 38.0)	35.0 (34.0, 37.0)	<0.001
TTD	55.0 (53.0, 58.0)	54.0 (51.0, 56.0)	<0.001	55.0 (52.0, 57.0)	53.0 (51.0, 56.0)	<0.001
APTD	57.0(54.0, 60.0)	55.0 (53.0, 58.0)	<0.001	56.0 (54.0, 59.0)	55.0 (52.0, 57.0)	<0.001
AFV	49 (43.0, 55.0)	48 (42.0, 53.0)	0.002	50 (45.0, 55.0)	48 (43.0, 55.0)	0.010
Pl thickness	26.0 (23.0, 28.0)	25.0 (23.0, 28.0)	0.012	26.0 (23.0, 28.0)	25.0 (23.0, 28.0)	0.002
UA-S/D	3.20 (2.85, 3.60)	3.30 (2.91, 3.80)	<0.001	3.19 (2.81, 3.60)	3.29 (2.91, 3.70)	<0.001
UA-PI	1.13 (1.02, 1.24)	1.17 (1.05, 1.28)	<0.001	1.12 (1.01, 1.24)	1.16 (1.04, 1.28)	<0.001
UA-RI	0.69 (0.65, 0.72)	0.70 (0.66, 0.74)	<0.001	0.68 (0.65, 0.72)	0.69 (0.66, 0.73)	<0.001
HBA1C	4.90 (4.80, 5.10)	4.90 (4.80, 5.10)	0.15	5.00 (4.80, 5.20)	4.90 (4.80, 5.10)	<0.001
FPG	4.20 (3.95, 4.47)	4.12 (3.87, 4.37)	<0.001	4.21 (3.97, 4.47)	4.11 (3.88, 4.34)	<0.001
GLU-1H	7.66 (6.77, 8.80)	7.50 (6.65, 8.54)	0.008	7.68 (6.62, 8.81)	7.62 (6.58, 8.77)	0.3
GLU-2H	6.46 (5.64, 7.41)	6.27 (5.49, 7.28)	0.014	6.47 (5.67, 7.43)	6.32 (5.44, 7.55)	0.043
TC	4.47 (4.01, 4.96)	4.39 (3.95, 4.88)	0.054	4.43 (4.01, 4.91)	4.46 (4.06, 4.95)	0.3
TG	1.27 (1.01, 1.63)	1.19 (0.96, 1.52)	<0.001	1.30 (1.03, 1.66)	1.27 (1.01, 1.59)	0.053
HDL	1.91 (1.65, 2.17)	1.93 (1.67, 2.17)	0.4	1.96 (1.70, 2.25)	2.02 (1.73, 2.31)	0.014
LDL	2.59 (2.16, 3.05)	2.53 (2.12, 2.98)	0.070	2.45 (2.08, 2.86)	2.44 (2.10, 2.90)	0.4

SUA, single umbilical artery; BPD, biparietal diameter; AC, abdominal circumference; HC, head circumference; FL, fetal length; HL, humeral length; TTD, transverse trunk diameter; APTD, anteroposterior trunk diameter; AFV; Pl thickness, placenta thickness; UA-S/D, umbilical artery systolic/diastolic ratio; UA-PI, umbilical artery pulsatility index; UA-RI, umbilical artery resistance index; HBA1C, glycosylated hemoglobin; FBG, fasting blood glucose; GLU-1H, one-hour glucose; GLU-2H, 2 h glucose; TC, total cholesterol; TG, triglycerides; HDL, high-density lipoprotein; LDL, low-density lipoprotein; SGA, small for gestational age. ^1^ Median (IQR); n (%). ^2^ Wilcoxon rank sum test; Pearson’s chi-squared test; Fisher’s exact probability test.

**Table 3:** Factors associated with SGA in women at the International Peace Maternity and Child Health Hospital (n = 11,746).

Characteristic	OR ^1^	95% CI ^2^	*p*-Value
Age			
<35	—	—	
≥35	1.23	(0.94, 1.59)	0.12
Gravidity			
1	—	—	
2	0.73	0.56, 0.93	0.014
≥3	0.62	0.43, 0.86	0.006
Parity			
1	—	—	
≥2	0.69	0.51, 0.95	0.020
BMI	0.89	0.85, 0.92	<0.001
GA	0.86	0.82, 0.91	<0.001
SUA			
No	—	—	
Yes	3.25	0.94, 8.61	0.033
AC	0.98	0.96, 0.99	0.012
HL	0.92	0.87, 0.98	0.004
APTD	0.98	0.93, 1.03	0.4
UA-S/D	1.40	1.20, 1.64	<0.001
FPG	0.70	0.55, 0.88	0.003

BMI, body mass index; GA, gestational age; SUA, single umbilical artery; AC, abdominal circumference; HL, humeral length; APTD, anteroposterior trunk diameter; UA-S/D, umbilical artery systolic/diastolic ratio; FBG, fasting blood glucose. ^1^ OR Odds ratio. ^2^ CI Confidence interval.

## Data Availability

Further data inquiries can be directed to the corresponding author.

## Supplementary Materials

The following supporting information can be downloaded at: https://www.mdpi.com/article/10.3390/jcm12082993/s1.
