# Supplementary material for: Development and Validation of a Small for Gestational Age Screening Model at 21–24 Weeks Based on the Real-World Clinical Data
Source: J Clin Med. 2023 Apr 20;12(8):2993. doi: 10.3390/jcm12082993 (PMC10142638; doi:10.3390/jcm12082993)
Supplement: Supplementary file 1 [file jcm-12-02993-s001.zip › jcm-2180198-supplementary.pdf]

Supplemental Material

Table S1 The TRIPOD Checklist: Prediction Model Development

Abbreviations: NA, not applicable

Section/Topic	Item	Checklist Item	Page
**Title and abstract**			
Title	1	Identify the study as developing and/or validating a multivariable prediction model, the target population, and the outcome to be predicted.	Title
Abstract	2	Provide a summary of objectives, study design, setting, participants, sample size, predictors, outcome, statistical analysis, results, and conclusions.	Abstract
**Introduction**			
Background and objectives	3a	Explain the medical context (including whether diagnostic or prognostic) and rationale for developing or validating the multivariable prediction model, including references to existing models.	Introduction, para 1-2
	3b	Specify the objectives, including whether the study describes the development or validation of the model or both.	Introduction, para 3
**Methods**			
Source of data	4a	Describe the study design or source of data (e.g., randomized trial, cohort, or registry data), separately for the development and validation data sets, if applicable.	Methods: Study population
	4b	Specify the key study dates, including start of accrual; end of accrual; and, if applicable, end of follow-up.	Methods: Study population
Participants	5a	Specify key elements of the study setting (e.g., primary care, secondary care, general population) including number and location of centers.	Methods: Study population
	5b	Describe eligibility criteria for participants.	Methods; Study population
	5c	Give details of treatments received, if relevant.	NA
Outcome	6a	Clearly define the outcome that is predicted by the prediction model, including how and when assessed.	Methods； Outcomes
	6b	Report any actions to blind assessment of the outcome to be predicted.	NA
Predictors	7a	Clearly define all predictors used in developing or validating the multivariable prediction model, including how and when they were measured.	Methods Variables measurements Supplementary Table S2
	7b	Report any actions to blind assessment of predictors for the outcome and other predictors.	Methods Variables measurements
Sample size	8	Explain how the study size was arrived at.	Methods; Study population

Missing data	9	Describe how missing data were handled (e.g., complete-case analysis, single imputation, multiple imputation) with details of any imputation method.	Methods<br>Data Processing para 2
Statistical analysis methods	10a	Describe how predictors were handled in the analyses.	Methods<br>Data Processing<br>Supplementary Table S2
	10b	Specify type of model, all model-building procedures (including any predictor selection), and method for internal validation.	Methods<br>Statistical analysis
	10d	Specify all measures used to assess model performance and, if relevant, to compare multiple models.	Methods<br>Statistical analysis
Risk groups	11	Provide details on how risk groups were created, if done.	Methods<br>Statistical analysis
**Results**			
Participants	13a	Describe the flow of participants through the study, including the number of participants with and without the outcome and, if applicable, a summary of the follow-up time. A diagram may be helpful.	Results<br>Part 1, pare 1<br>Figure 1
	13b	Describe the characteristics of the participants (basic demographics, clinical features, available predictors), including the number of participants with missing data for predictors and outcome.	Results<br>Part 1, pare 2<br>Table 1 and 2
Model development	14a	Specify the number of participants and outcome events in each analysis.	Results<br>Part 1, pare 1
	14b	If done, report the unadjusted association between each candidate predictor and outcome.	Table S4
Model specification	15a	Present the full prediction model to allow predictions for individuals (i.e., all regression coefficients, and model intercept or baseline survival at a given time point).	Results<br>Part 2<br>Table 3
	15b	Explain how to the use the prediction model.	Results<br>Part 3<br>Figure 2
Model performance	16	Report performance measures (with CIs) for the prediction model.	Results<br>Part 3
**Discussion**			
Limitations	18	Discuss any limitations of the study (such as nonrepresentative sample, few events per predictor, missing data).	Discussion pare 6
Interpretation	19b	Give an overall interpretation of the results, considering objectives, limitations, and results from similar studies, and other relevant evidence.	Discussion section
Implications	20	Discuss the potential clinical use of the model and implications for future research.	Discussion section

Other information			
Supplementary information	21	Provide information about the availability of supplementary resources, such as study protocol, Web calculator, and data sets.	Supplementary information
Funding	22	Give the source of funding and the role of the funders for the present study.	Acknowledgements section

Table S2 Variables names, variables types, variables descriptions, along with their categorizations.

variable	Variable type	variable meaning	Descriptions or categorizations
Age	Numeric	maternal age	(i) < 35y and (ii)⩾35y(i)
Hus age	Numeric	Husband’s age	
Gravida	Numeric	gravidity	(i)=1， (ii)=2 and (iii)⩾3
Parity	Numeric	Parity at sterilization	(i)=1 and (ii)⩾2
Edu	Numeric	maternal educational level	(i)Bachelor, (ii)Above bachelor’s and (iii)Below bachelor’s
Hus edu	Numeric	Husband’s educational level	
BMI((kg/m2)	Numeric	pre-pregnancy body mass index	underweight ＜18.5， normal weight 18.5–24.9， overweight 25.0–29.9 and obesity 30.
Smoking	Numeric	Smoking history before pregnancy	(i)=yes and (ii)⩾no
Alcohol	Numeric	Alcohol history before pregnancy	(i)=yes and (ii)⩾no
Family history	Numeric	Family History of Diabetes or hypertension	(i)=yes and (ii)⩾no
GA	Continuous	Gestational age	-
SBP	Continuous	systolic blood pressure	around 24 weeks gestational
DBP	Continuous	diastolic blood pressure	
AC	Continuous	fetal abdominal circumference	sonographic measurements all between 21-24 weeks
BPD	Continuous	fetal biparietal diameter	
HC	Continuous	fetal head circumference	
FL	Continuous	fetal femur length	
HL	Continuous	fetal humerus length	
TTD	Continuous	fetal abdominal transverse trunk diameter	
APTD	Continuous	fetal abdominal anteroposterior trunk diameter	
AFV	Continuous	maximum amniotic fluid volume	
UA-S/D	Continuous	systolic/diastolic ratio (S/D)	Umbilical artery Doppler flow
UA-PI	Continuous	pulsatility index	
UA-RI	Continuous	resistance index	
placental thickness	Continuous	placental thickness	-
Placental location	Numeric	Placental location	normal, placenta previa or low-lying placenta
placental sinusoids	Numeric	placental sinusoids	(i)=yes and (ii)⩾no
SUA	Numeric	single umbilical artery	(i)=yes and (ii)⩾no
velamentous placenta	Numeric	velamentous placenta	(i)=yes and (ii)⩾no
TC	Continuous	total cholesterol	between 9 and 13 weeks of gestation
TG	Continuous	triglycerides	
HDL	Continuous	high-density lipoprotein	
LDL	Continuous	low-density lipoprotein	
HBA1C	Continuous	hemoglobin	75-g oral glucose tolerance test (OGTT) between gestational weeks 24 and 28
FPG	Continuous	fasting plasma glucose	
GLU-1H	Continuous	one-hour glucose	

GLU-2H	Continuous	two-hour glucose	

1

Table S3 The frequency and percentage of variables missing values

Characteristic	Missing Frequency	Percentage
UA-S/D	1536	6.40%
UA-PI	1525	6.36%
UA-RI	1527	6.36%
HBA1C	1388	5.78%
FPG	1031	4.30%
GLU-2H	975	4.06%
GLU-1H	1073	4.47%
TC	5648	23.54%
TG	5642	23.51%
HDL	5646	23.53%
LDL	5646	23.53%

Table S4 Comparison of the coefficient (β), standard errors (SE), and p-values between complete case analysis and missing values handling for the multivariate logistics regression model.

Characteristic	Complete Case Analysis (N=14006)			Missing Values Handling (N=23783)		
	β	SE	p-value	β	SE	p-value
Age (≥35)	0.243	0.122	0.047	0.208	0.134	0.120
Gravidity (2)	-0.183	0.117	0.119	-0.317	0.128	0.014
Gravidity (≥3)	-0.281	0.165	0.089	-0.485	0.176	0.006
Parity (≥2)	-0.423	0.147	0.004	-0.365	0.157	0.020
BMI	-0.123	0.019	<0.001	-0.120	0.020	<0.001
GA	-0.136	0.028	<0.001	-0.149	0.029	<0.001
SUA (Yes)	0.287	0.747	0.701	1.178	0.551	0.033
AC	-0.040	0.009	<0.001	-0.024	0.009	0.012
HL	-0.098	0.027	<0.001	-0.081	0.028	0.004
APTD	-0.021	0.022	0.324	-0.021	0.024	0.369
UA-S/D	0.293	0.076	<0.001	0.338	0.079	<0.001
FPG	-0.389	0.116	<0.001	-0.362	0.123	0.003

Table S5 Univariate analysis in the training set

Characteristic	OR[1]	95% CI[2]	p-value
Age (≥35)	0.79	(0.62, 1)	0.05
Edu (Above bachelor's)	1.02	(0.81, 1.29)	0.86
Edu (Below bachelor's)	0.94	(0.75, 1.18)	0.59
Edu (Unknown)	1.04	(0.62, 1.75)	0.88
Hus edu (Above bachelor's)	0.98	(0.78, 1.23)	0.83
Hus edu (Below bachelor's)	0.84	(0.66, 1.07)	0.16
Hus edu (Unknown)	0.97	(0.66, 1.42)	0.87
Hus age (≥35)	0.76	(0.62, 0.93)	0.01
Gravidity (2)	0.62	(0.5, 0.77)	<0.001
Gravidity (≥3)	0.47	(0.36, 0.61)	<0.001
Parity (≥2)	0.51	(0.41, 0.64)	<0.001
Smoking (Yes)	1.28	(0.4, 4.1)	0.68
Smoking (Unknown)	0.80	(0.46, 1.4)	0.43
Alcohol (Yes)	0.62	(0.25, 1.51)	0.29
Alcohol (Unknown)	0.79	(0.45, 1.39)	0.42
BMI	0.87	(0.84, 0.9)	<0.001
Family History (Yes)	0.93	(0.74, 1.17)	0.55
Family History (Unknown)	0.95	(0.57, 1.59)	0.86
SYFS (Assisted reproduction)	0.99	(0.7, 1.42)	0.97
SYFS (Unknown)	0.94	(0.52, 1.68)	0.83

1 ***OR*** **Odds ratio**
2 ***CI***

GA	0.91	(0.86, 0.96)	<0.001
SBP	1.00	(0.99, 1.01)	0.97
DBP	1.01	(1, 1.02)	0.22
Placental location (low-lying placenta)	0.59	(0.24, 1.45)	0.25
Placental location (placenta previa)	0.68	(0.32, 1.44)	0.31
Velamentous placenta (Yes)	0.70	(0.1, 5.13)	0.73
Placental sinusoids (Yes)	0.43	(0.06, 3.09)	0.4
SUA (Yes)	3.21	(1.12, 9.16)	0.03
BPD	0.91	(0.88, 0.94)	<0.001
AC	0.96	(0.95, 0.97)	<0.001
HC	0.97	0.55, 0.88	0.003
FL	0.87	(0.96, 0.98)	<0.001
HL	0.83	(0.84, 0.91)	<0.001
TTD	0.92	(0.8, 0.87)	<0.001
APTD	0.90	(0.9, 0.94)	<0.001
AFV	0.98	(0.97, 0.99)	<0.001
Placental thickness	0.97	(0.94, 0.99)	0.01
UA-S/D	1.47	(1.27, 1.72)	<0.001
UA-PI	3.35	(1.91, 5.88)	<0.001
UA-RI	53.78	(9.37, 308.55)	<0.001
HBA1C	0.84	(0.62, 1.14)	0.27
FPG	0.55	(0.43, 0.7)	<0.001

GLU-2H	0.93	(0.87, 1)	0.04
GLU-1H	0.92	(0.86, 0.98)	0.01
TC	0.90	(0.79, 1.02)	0.09
TG	0.73	(0.61, 0.89)	<0.001
HDL	1.14	(0.91, 1.43)	0.24
LDL	0.87	(0.76, 1)	0.05

Confidence interval
